# Role of regular medical visits in mitigating increased suicide risk during the early COVID-19 pandemic in Kobe, Japan

**DOI:** 10.1186/s12875-025-02707-2

**Published:** 2025-01-23

**Authors:** Daisuke Miyamori, Yasushi Nagasaki, Shuhei Yoshida, Saori Kashima, Wataru Omori, Kei Itagaki, Masanori Ito

**Affiliations:** 1 Department of General Internal Medicine, Hiroshima University Hospital, 1-2-3, Kasumi, Minami-ku, Hiroshima-shi, Hiroshima-ken, 734-8551 Japan; 2 Hyogo Medical Examiner’s Office, Hyogo, Japan; 3 Graduate School for International Development and Cooperation, Hiroshima University, Hiroshima, Japan; 4 Environmental Health Sciences Laboratory, Graduate School of Advanced Science and Engineering, Hiroshima University, Hiroshima, Japan; 5 Department of Psychiatry and Neurosciences, Graduate School of Biomedical Sciences, Hiroshima University, Hiroshima, Japan

**Keywords:** Suicide, COVID-19, Japan, Adolescent, Primary care, Psychiatric department, Interrupted time series analysis, Population database

## Abstract

**Background:**

Japan has one of the lowest COVID-19 death rates, while the annual suicide rate in 2020 has risen for the first time since 2007. This study aimed to identify high-risk populations and assess the impact of medical visits on suicide trends post-COVID-19 pandemic in Japan.

**Method:**

This quasi-experimental study analyzed a population-based database from Hyogo Prefecture (2012–2022). Interrupted time-series analyses identified level and trend changes in monthly suicide rates per 1 million population during the exposure period (2020–2022) versus the control period (2012–2019). Regular visits to primary care and psychiatry stratified analysis.

**Results:**

2181 cases were analyzed, with two-thirds male and a median age of 54. Primary care physicians and psychiatric history were present in 69% and 40% of patients. The study found significant level changes in suicide rates overall (4.14, 95% CI: 1.70, 6.58) among individuals without regular primary care physician visits (2.83, 95% CI: 1.35, 4.32) and without psychiatric visits (2.85, 95% CI: 0.56, 5.14). In contrast, no significant changes were observed in individuals with regular primary care (0.99, 95% CI: -0.78, 2.76) or regular psychiatric visits (0.59, 95% CI: -0.98, 2.16). The trend changes were not significant in any of the groups.

**Conclusion:**

This study suggests that a history of attending a medical institution may have prevented the rapid increase in suicides during the early stages of the COVID-19 pandemic.

**Supplementary Information:**

The online version contains supplementary material available at 10.1186/s12875-025-02707-2.

## Introduction

Japan has one of the highest suicide rates among developed nations, with an average of 20,000 deaths annually [1, 2]. Notably, young people and older men are disproportionately affected. Existing factors contributing to this concern include hesitation and the societal stigma surrounding seeking help from mental health resources [3]. Furthermore, the limited involvement of primary care physicians in managing mental illnesses has long been recognized [4]. This confluence of factors underscores the seriousness of Japan’s suicide problem, which the COVID-19 pandemic may further exacerbate.

Indeed, 2020 witnessed a 15% increase in suicides compared to the previous year, marking the first such increase in 11 years [5, 6]. This increase was particularly pronounced among younger individuals and women [7]. Despite this alarming trend, few studies have examined the pandemic’s short- and long-term impact on suicide trends. However, while there is an examination of risk for variations in suicide risk with respect to age and gender, the impact of other factors on the vulnerable population has not been examined.

The COVID-19 pandemic has significantly affected global health, with over six million deaths reported worldwide. Japan was one of the first countries to be affected, experiencing its first case in January 2020. The swift spread of the virus prompted the Japanese government to implement measures, such as border closures, social distancing, and mask mandates [8, 9]. These initiatives and high vaccination rates have enabled Japan to combat successive waves of infection and achieve one of the lowest cases and mortality rates globally [10]. However, the pandemic has also triggered substantial behavioral changes, including reduced non-essential travel, increased social isolation, and adherence to mask mandates. These factors, in turn, have contributed to economic hardship, social isolation, and an increase in mental health hospitalization and suicide [11, 12]. The COVID-19 pandemic has influenced the frequency of primary care visits, with a general trend of reduced in-person visits [13]. The function of primary care physicians and the impact of psychiatry department visits on suicide risk during the pandemic remain unclear. Given the physical barrier created by policies discouraging non-essential outings, it is crucial to determine the role that regular medical institution visits play in providing informed policy recommendations.

Therefore, this study aimed to identify high-risk populations and assess the impact of medical visits on suicide trends in Japan following the COVID-19 pandemic using real-world data. By doing so, we aim to reveal discrepancies between the supply and demand for mental health services during times of crisis, informing the development of efficient intervention methods and clarifying the role of medical institutions in mitigating the mental health consequences of future pandemics. Learning from the pandemic’s impact on mental health is crucial for building resilience and preventing similar crises in the future.

## Methods

### Design

We conducted a quasi-experimental study using an interrupted time-series analysis.

### Data and population

The Hyogo Medical Examiner’s Office conducts inquests and medical examinations per the Autopsy Preservation Act of Kobe City [14]. This center covers all unnatural deaths, including all the suspicious suicides, injuries, and non-endogenous cases, in Kobe City except for northern and western districts. The cases investigated in the office accounted for 10.6% of all death cases in the targeted area [15]. The office covers approximately 1.05 million people, and the age distribution (11.8% under 15, 59.4% 15–64, 28.9% 65 and over) is similar to Japan’s demographics (11.9% under 15, 59.5% 15–64, 28.6% 65 and over) [16]. Specifically, the Hyogo Medical Examiner’s Office performs autopsies and examinations of corpses to determine the cause of death of persons whose deaths occurred in Kobe. The information obtained during autopsies is used in clinical and preventive medicine to contribute to Public Health. Approximately 70% of the patients underwent an autopsy with toxicological investigation.

### Main outcome

The main outcome of this study was the monthly suicide rate per 1 million population in Kobe City, Japan. It divided the monthly number of suicide cases by the total population in the target area each month. Data from January 2011 to December 2022 were collected and analyzed.

### Variables for baseline and subgroup analysis

In our study, baseline variables included demographic and clinical factors. The data included age, sex, activity of daily living (ADL), living alone or not, and visiting status of the primary care and psychiatric department of each suicide victim. ADL was dichotomized as independent and non-independent. The presence or absence of regular visits to primary care physicians and psychiatric departments were also obtained. This information is verified by police on-site inspections and environmental investigations, including family registers, interviews with family members, and interviews with hospitals. A patient was considered to have regular visits if he or she visited the primary care clinic or psychiatry department consistently, ranging from once a month to several times a year.

### Statistical analysis

We used an interrupted time-series (ITS) analysis to examine the impact of the COVID-19 pandemic on suicide rates [17]. ITS analysis is a quasi-experimental design used to evaluate the impact of interventions or exposures that occur at a particular point in time. It is particularly useful for evaluating interventions in public health, as it can estimate the impact of interventions on real-world outcomes.

ITS analysis assumes that the outcome variable follows a predictable trend in the absence of intervention. The impact of the intervention was estimated by comparing the actual trend in the outcome variable after the intervention with the predicted trend. ITS analysis has several strengths that make it a valuable tool for evaluating the impact of interventions on public health [18]. ITS analysis can be used to evaluate the impact of interventions and exposures in real-world settings.

$$\eqalign{& \>y\> = \>b0\> + \>b1\>*\>time\> + \>b2\>*\>exposure \cr & \> + b3*\left( {time*exposure} \right) + \>e \cr}$$

y​ represents the outcome at the time, *b0* is the intercept indicating the baseline level of the outcome before the intervention, and *b1* represents the slope of the outcome over time before exposure. Variable *b2* captures the immediate level change in the outcome following the exposure. At the same time, *b3*, the interaction term, represents the change in the slope of the outcome after the exposure compared to the pre-intervention period.

We define the pandemic period from January 2020 to December 2022 as exposure. As the outcome variable is a ratio (suicide rates per 1 million population), we used an Ordinary Least Squares (OLS) regression model with Newey-West standard errors. We used ITS analysis to estimate the level change in suicide rates after the COVID-19 pandemic, as well as the changes in trends between the pre-pandemic and pandemic periods. We also conducted a subgroup analysis to identify populations at a considerable risk of suicide during the pandemic. We stratified the data by regular visits to psychiatry hospitals and primary care physicians.

Subsequently, we conducted sensitivity analyses for the main and subgroup analyses using the different cutoffs of the pre-pandemic and pandemic periods: December 2019 to March 2020 were set as implementation terms and excluded from the analysis. The details of the sensitivity analysis are described in the Supplementary material. In this study, a p-value less than 0.05 is considered statistically significant. All statistical analyses were performed using the STATA 18 MP software (Stata Corp, TX, USA).

## Results

**Fig. 1:**
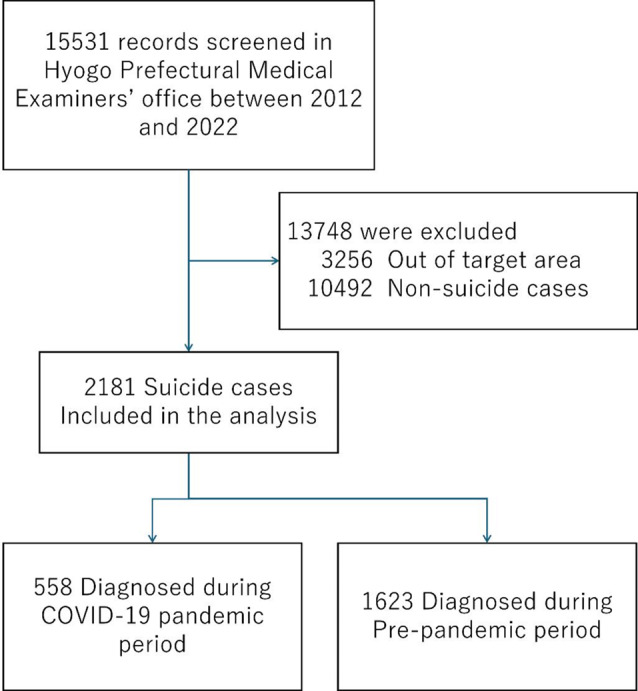
Flow chart of case selection

Figure 1 shows the flowchart of the cases. Among 15,531 unnatural death cases were included in this study. After excluding 3256 cases whose addresses were out of the sampling regions and 10,492 cases whose causes of death were out of suicide, 2181 suicide cases were included, consisting of 558 cases and 1623 cases for the pandemic and pre-pandemic periods, respectively.

**Table 1:** Baseline characteristics of suicide cases

	Total	Pre-pandemic	Pandemic
	*N* = 2,181	*N* = 1,623	*N* = 558
Age, Median (IQR)	54 (40–70)	55 (40–69)	53 (40–70)
Male, N (%)	1,438 (66%)	1,079 (66%)	359 (64%)
Primary care physician, N (%)	1,513 (69%)	1,128 (70%)	385 (69%)
Psychiatry visit, N (%)	870 (40%)	654 (40%)	216 (39%)
Living Alone, N (%)	907 (42%)	670 (41%)	237 (42%)
Independent ADL, N (%)	2,074 (95%)	1,541 (95%)	533 (96%)

In cases of deaths due to multiple reasons, all reasons are counted. N, number; IQR, inter-quartile range; ADL, activity daily living

Table 1 shows the baseline patient characteristics. Of the included patients, 1438 (66%) were male, with a median (inter-quartile range) age of 54 years (40–70). Single-person households were found in 907 cases (42%), the presence of a primary care physician was observed in 1513 cases (69%), and 870 cases (40%) had attended a psychiatric hospital. ADLs were independent in more than 95% of patients, and no major changes were observed before or after the COVID pandemic.

**Fig. 2:**
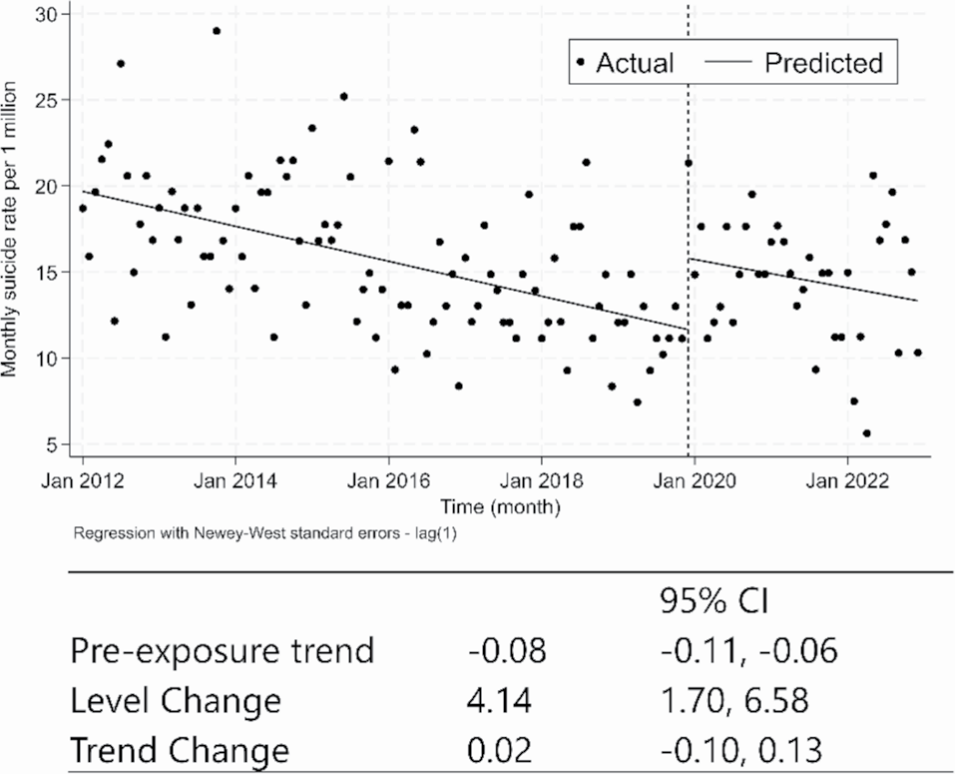
Result of Interrupted time series analysis before and during the COVID-19 pandemic The data show trends in the monthly number of suicide rates from January 2012 to December 2022 in Kobe, Japan. Solid lines indicate approximate lines for the exposure and target periods, and each plot shows the actual monthly suicide rate per 1 million population. The difference in intercepts before and during the pandemic was analyzed as a level change, whereas the difference in trends before and during the pandemic was analyzed as a trend change

The results of the main analysis are shown in Fig. 2. ITS analysis showed that there was a significant level increase in monthly suicide rates per 1 million population in Kobe City during the COVID-19 pandemic (level change; 4.14, 95% confidence interval [CI]:1.70–6.58). There was no significant decrease in the trend of the trend in suicide rates after the pandemic (trend change; 0.02, 95%CI: -0.10, 0.13). In the sensitivity analysis, level change and trend change are 3.36 (95% CI: 0.88, 5.83) and 0.05 (95% CI: -0.07, 0.18) after excluding the 3-month implementation term (Supplementary Fig. 1).

**Fig. 3:**
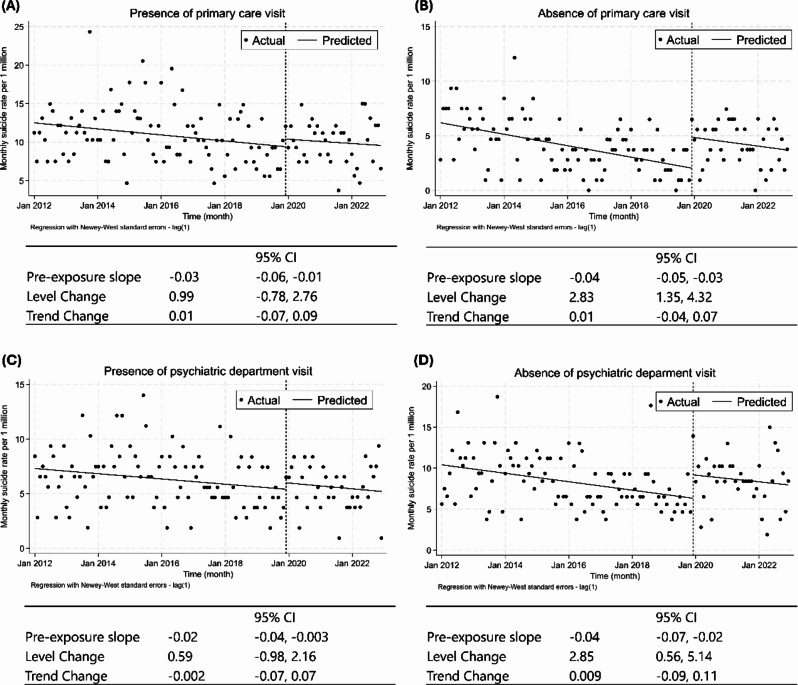
ITS analysis before and during the COVID-19 pandemic for the presence and absence of regular medical visit Interrupted time series analyses are shown for the (**A**) presence and (**B**) absence of primary care visits, and (**C**) presence and (**D**) absence of psychiatric department visits with level and trend changes. The level change represents the intercept, an increase or decrease from December 2019 to January 2020, distinct from the ongoing trend. A value greater than zero indicates an immediate increase in the suicide rates during the pandemic. The trend change represents the difference in trends before and during the COVID-19 pandemic. A value greater than zero indicated an increase in the trend of suicide rates during the pandemic. ITS, interrupted time series

The subgroup analysis showed that the increase in suicide rates after the COVID-19 pandemic was particularly pronounced among those who did not have access to mental health care or primary care physicians (Fig. 3). The level change in suicide rates was also 2.85 (0.56, 5.14) cases per month among people who had not visited a psychiatry hospital in the year before their death, compared to 0.59 (-0.98, 2.16) cases per month among people who had visited a psychiatry department. The level change in suicide rates was also 2.83 (1.35, 4.32) cases per month among people who had not regularly visited primary care, compared to 0.99 (-0.78, 2.76) cases per month among people who had regularly visited primary care. In the sensitivity analyses, the level change among people who had not regularly visited psychiatric departments and primary care facilities was 2.24 (95% CI: -0.15, 4.63) and 2.59 (95% CI: 0.87, 4.31), respectively, while the level changes were 0.28 (-1.43, 1.99) and 0.73 (-1.23, 2.70) for those who had regularly visited psychiatric departments and primary care facilities (Supplementary Fig. 2).

## Discussion

Our findings suggest that the COVID-19 pandemic has had a significant impact on suicide rates in Kobe City, Japan. The subgroup analysis showed that the increase in suicide rates was particularly pronounced among younger people and those who were absence of regular access to mental health care or primary care physicians.

The results of this study indicated that the increase in suicide after the COVID-19 pandemic appeared promptly, and the increase was significant in younger populations and in populations without regular visits to the psychiatric department or primary care. The findings of our study are consistent with those of other studies showed suicide rates increased in countries, including the United States, Canada, and Japan [19]. Other studies have shown a decreased trend during the 1st wave and an increase during 2nd wave, which is discrepant from the current study [6]. Kobe, the focal area of this study, is the sixth largest city in Japan, and previous studies have demonstrated that the risk of anxiety disorders in the early stages of the COVID-19 epidemic was higher in urban areas [20]. Furthermore, given that the risk of suicide is lower in urban areas compared to rural areas, it is plausible that the burden of behavioral changes induced by COVID-19 is more pronounced in urban settings, potentially explaining the sharp increase in suicide risk observed in this study [21]. In this study, the monthly suicide numbers increased at the beginning of the pandemic, a time when COVID-19 had barely appeared in Hyogo Prefecture. However, the trend changes were insignificant overall or in each subgroup category. These results suggest that the perspective of anxiety about COVID-19 infection, stress from uncertain medical information, and pessimism about the future, rather than the COVID-19 incidence itself, may have had a major influence [22, 23]. 

Previous studies have found that the number of patients visiting psychiatry departments in Japan increased by 15 to 20% during the pandemic, and 80% of psychiatry departments experienced overcrowding [24–26]. This might result in a decreased quality of care [27]. This is likely due to several factors, including increased demand for mental health care, decreased availability of mental health services, and financial barriers to mental health care. Another factor is stigma and lack of public education about mental health. People with mental illness are often unaware of the signs and symptoms of mental health problems or the importance of seeking professional help [28]. People who struggle with mental health problems or other difficulties may feel ashamed to seek help, as they may not want to burden their families or communities [29]. The combination of increasing mental illness and psychiatric overcrowding, behavioral restraints limiting access to medical care, and hesitation to seek care due to stigma may have contributed to an increase in suicides, the worst consequence. The scoping review has also shown other risk factors for suicidal behavior during the COVID-19 pandemic to be risk factors for suicidal behavior in patients with pre-existing mental illness, with particular recommendations for social isolation, loneliness, limited access, substance use disorders, and alcohol abuse. The review also found that patients with existing mental illnesses are at risk for suicidal behavior [30]. It is important to invest in mental health services and address the financial barriers to mental health care to improve access to care for people in Japan.

A supplemental analysis excluding the implementation term between December 2019 and March 2020 showed similar trends to the main analysis. These results support the robustness of the main findings, indicating that the COVID-19 pandemic was associated with an immediate increase in suicide rates. At the same time, the long-term trend did not significantly change. The consistency between the main and supplemental analyses strengthens the conclusion that the pandemic had an immediate impact on suicide rates in Kobe City, Japan.

It is also suggested that primary care visits may have acted as a buffer against the impact of the early stages of the COVID-19 pandemic. Japan has very good accessibility to medical facilities, and visits are several times more frequent than in other countries [31]. However, in various countries, including Japan, behavioral changes have led to an extreme decrease in visits to healthcare facilities in the early stages of the COVID-19 pandemic [32]. Although this could be due to a decline in actual medical demand due to a decrease in infections other than COVID-19, particularly in the early stages of the pandemic, uncertainties about infection and restrictions on government action may have had a greater impact [33, 34]. Although telemedicine is temporarily permitted, reduced opportunities for face-to-face medical care trigger reduced verbal and nonverbal medical communication opportunities. Patients with regular outpatient visits probably have the opportunity to discuss their psychiatric symptoms. However, it was assumed that in the population without regular outpatient visits, reduced opportunities to connect to medical care may have failed to suppress the process that led to suicide.

The findings of this study suggest a potential reduction in suicide risk during the COVID-19 pandemic among patients who regularly visit medical institutions. This highlights the importance of maintaining contact with medical institutions beyond the scope of chronic disease treatment. Adolescence, a developmental period characterized by heightened susceptibility to change, typically exhibits a lower incidence of chronic disease and fewer routine hospital visits [35, 36]. To ensure continuity of medical care, the role of primary care physicians who provide care for common diseases across all ages is essential. During periods of notable change, such as the COVID-19 pandemic, generations more susceptible to anxieties can experience marked shifts in behavior. To mitigate these negative impacts, addressing their anxiety and negative perceptions is crucial.

In addition, in Japan, where the threshold for visiting a psychiatrist is high, it is said that those with mild conditions or low risk will visit primary care physicians first [37]. Although the frequency of treatment for psychosomatic diseases by primary care physicians in Japan has been estimated to be lower than that in other countries, our study suggests that primary care physicians play a vital role in reducing suicide [4]. Therefore, a more established training system for psychosomatic diseases and improvement in reimbursement for seeing mental illness enabled primary care physicians to strengthen the efficiency in mental health in the future [38]. Amid overcrowded psychiatric care and highly frequent outpatient in primary care, intervention for mental illness by primary care physicians is an initiative that can improve these situations, including policies to reduce the extremely frequent medical visits in primary care and sufficient reimbursement to ensure that time is allocated to patients who need priority care.

The strength of this study is that it used a population-based database, and the demographic characteristics in the sampling area are similar to those of Japan. In addition, as this study used real-world data, it is considered to have a high degree of generalizability.

On the other hand, the limitations of this study include: (1) lack of accurate data on the population without regular primary care or psychiatric visits during the exposure period, (2) the possibility of misclassification of suicide, and (3) the risk of mental illness caused by COVID-19 infection. First, the most important limitation of our subgroup analysis is the lack of accurate data on the population without regular primary care or psychiatric visits during the pandemic. This absence of a proper denominator limits the validity of our interpretation regarding the reported increases in suicide rates in this subgroup. Future studies should aim to collect more comprehensive data on healthcare utilization patterns to enable more robust subgroup analyses. Second, regarding the possibility of misclassification, it is considered that the possibility of misclassification is low because a certified forensic pathologist finally judged each case in this study after investigating the surrounding situation by neighbors, family, and medical professionals by police inspection divisions. The selection bias might be undeferential and, therefore, has a minor impact on the results. Finally, regarding the risk of mental illness caused by COVID-19, it is unknown whether each suicide victim had COVID-19. In the case of suicide cases in 2022, when the pandemic had spread to one-third of the population, the impact of mental illness caused by COVID-19 and the subsequent increase in suicide frequency, not behavioral change, could not be clarified in this study. However, COVID-19 was not prevalent in Hyogo Prefecture until the beginning of 2022; thus, it may have had a negligible impact on the level increase in this study.

## Conclusion

Using real-world data, this study estimated the short- and long-term effects of the COVID-19 pandemic on the frequency of suicide. It was also confirmed that the frequency of suicide has increased since the early stages of the pandemic. Additionally, the frequency of suicide was more pronounced among younger people. On the other hand, there was no sharp increase in the frequency of suicide among those with a history of visiting a psychiatrist or primary care physician. To address the increase in suicide frequency caused by behavioral changes during the COVID-19 pandemic, it is essential to take measures to connect patients to primary care more appropriately during peacetime and to improve the imbalance between the supply and insufficient demand of psychiatry.

## Electronic supplementary material

Below is the link to the electronic supplementary material.

Supplementary Material 1

## Data Availability

The dataset employed for analysis in this study is accessible from the Hyogo Medical Examiner’s office, although it is subject to licensing terms for the present investigation. Consequently, certain restrictions apply, and the data are not broadly accessible to the public. For inquiries regarding obtaining the dataset utilized in this study, kindly contact the corresponding author, DM.
