# Supplementary material for: Role of regular medical visits in mitigating increased suicide risk during the early COVID-19 pandemic in Kobe, Japan
Source: BMC Prim Care. 2025 Jan 23;26:18. doi: 10.1186/s12875-025-02707-2 (PMC11756082; doi:10.1186/s12875-025-02707-2)

**Title:** **Role of Regular Medical Visits in Mitigating Increased Suicide Risk During the Early COVID-19 Pandemic in Kobe, Japan**

Supplementary Files

Supplemental material.

To evaluate the impact of interventions and account for potential implementation and exposure effects, a sensitivity analysis was performed. The first intervention (T1​) was used as the start of the implementation period, while the second intervention (T2​) defined the start of the exposure period. The analysis focused on calculating the difference in outcome levels and trends between the pre-intervention period and the post-exposure period.

The outcome (y​) was modeled using a time variable (Time​), and two intervention indicators (Implementation and Exposure​), along with their interaction terms.

$$y = b0 + b1 * time + b2 *implementation +b3 \left( time*implementation \right)+ b4*exposure+b5 (time*exposure)+e$$

Where:

1. ***b0*:** The baseline level of the outcome at the start of the observation period.
2. ***b1*​:** The pre-intervention trend, representing the rate of change in the outcome over time before any intervention.
3. ***b2*​:** The immediate level change associated with the first intervention.
4. ***b3*​:** The change in the slope of the outcome after the start of implementation term.
5. ***b4*​:** The immediate level change associated with the exposure.
6. ***b5*​:** The change in the slope of the outcome after the second intervention.
7. ***e* ​:** The error term, capturing variability not explained by the model.

The level change at T2​ was calculated as the sum of the immediate level change during the implementation period (b2b_2b2​), the cumulative effect of the slope change during the implementation period (b3×ΔT, where ΔT=T2−T1​), and the immediate level change at the start of the exposure period (b4​). The trend change between the pre-intervention and post-exposure periods was calculated as the sum of the trend changes during the implementation and exposure periods (b3 + b5 ​). By accounting for both immediate and cumulative effects, this approach ensures a comprehensive evaluation of the interventions’ impact on outcome levels and trends over time.

$$trend change = b3 +b5$$

$$level change = b2+b4+b3*\Delta T$$

Supplementary Figure 1

Result of sensitivity analysis before and during COVID-19 pandemic

Data show the trends of monthly number of suicide cases from January 2012 to December 2022 in Kobe, Japan. Solid lines indicate approximate lines for the exposure and target periods; each plot shows the actual number of suicides per month. The term between December 2019 and March 2020 were excluded from the analysis as implementation term. Differences in intercept between before and during pandemic were analyzed as level change, while the difference in slope between before and during pandemic were analyzed as trend change.

Supplementary Figure 2

Sensitivity analysis for subgroup analysis

Interrupted time series analyses are shown for the (A) presence and (B) absence of primary care visit, and (C) presence and (D) absence of psychiatric department visit with level and trend changes. Level changes and trend changes were shown with 95% confidence interval. Level changes represent the intercept, an increase or decrease from December 2019 to March 2020 that is distinct from ongoing trend; more than zero indicates immediate increase the number of suicide cases during pandemic. Trend changes represent the difference in slope between pre- and during COVID-19 pandemic; more than zero indicates the increases in slope of suicide cases during pandemic.


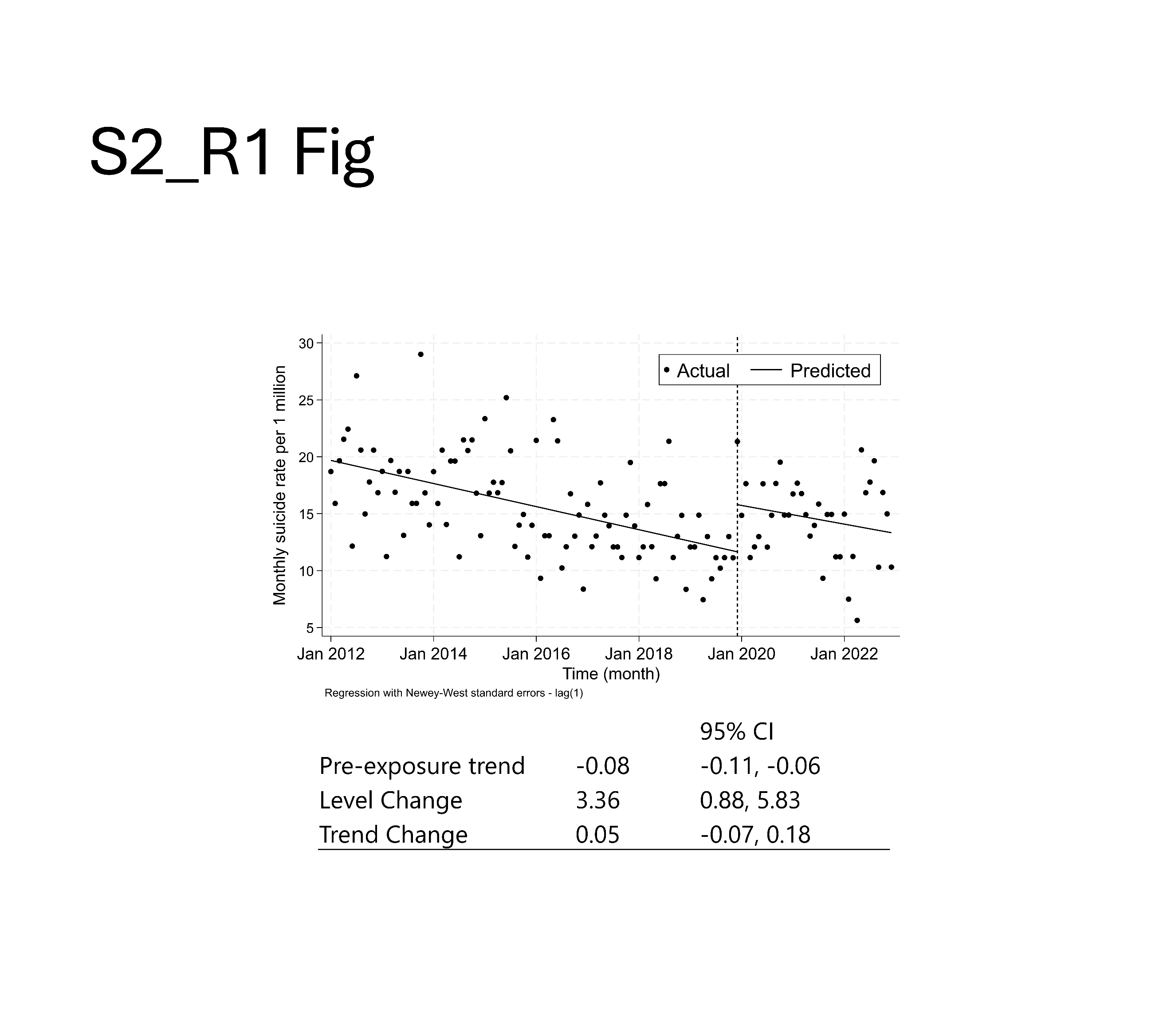

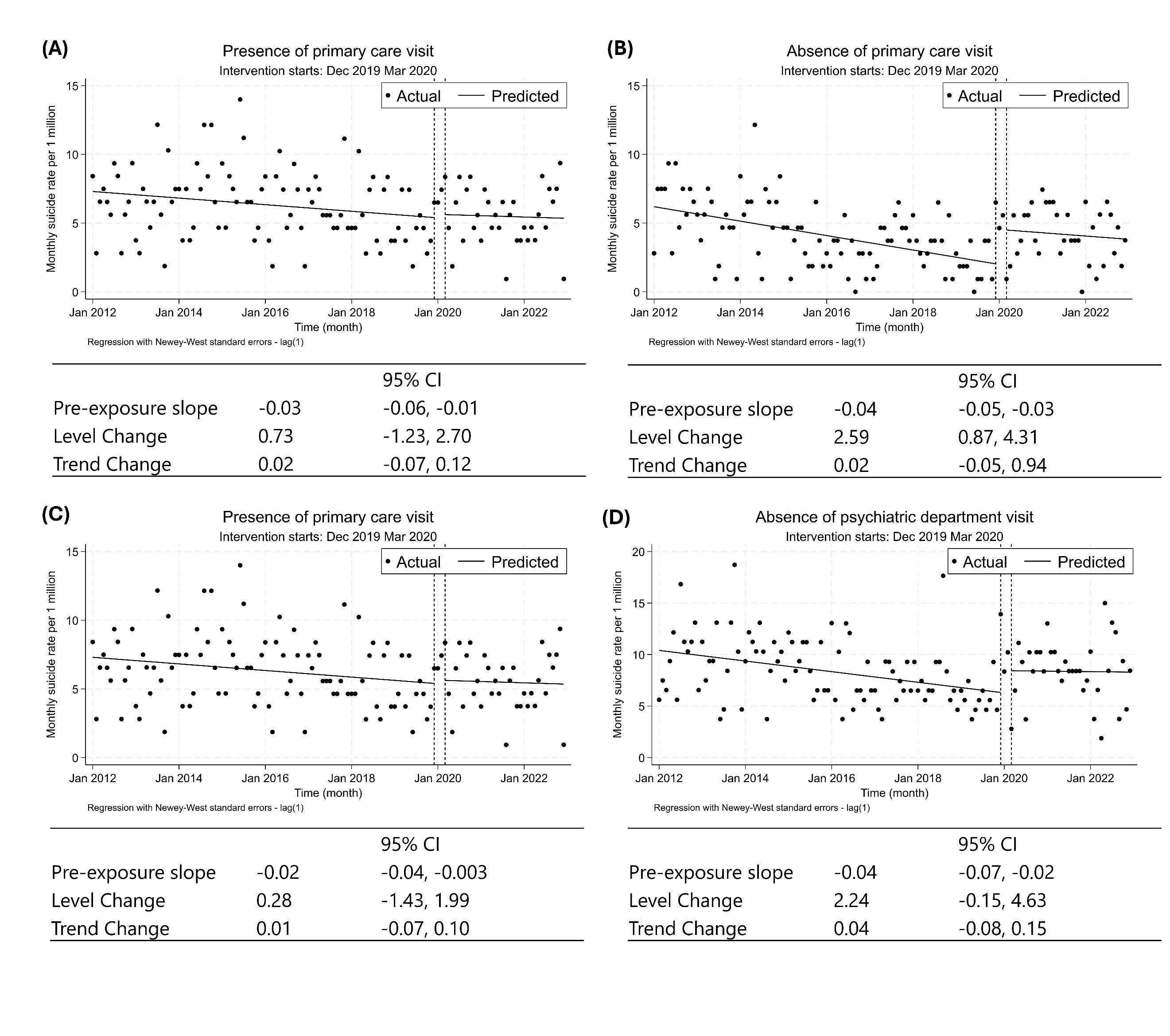

Supplement: Supplementary file 1 — Supplementary Material 1 [file 12875_2025_2707_MOESM1_ESM.docx]
